# Homology analysis between clinically isolated extraintestinal and enteral *Klebsiella pneumoniae* among neonates

**DOI:** 10.1186/s12866-020-02073-2

**Published:** 2021-01-11

**Authors:** Chun-mei Chen, Min Wang, Xian-ping Li, Peng-ling Li, Jing-jing Tian, Kan Zhang, Can Luo

**Affiliations:** 1 Department of Laboratory Medicine, The Second Xiangya Hospital, Central South University, 139 Renmin Road, Changsha, 410011 Hunan China; 2 Department of Laboratory Medicine, The Fifth Xiangya Hospital, Central South University, Changsha, Hunan China; 3 Department of Laboratory Medicine, Jiangxi Cancer Hospital, Nanchang, Jiangxi China

**Keywords:** *Klebsiella pneumoniae*, Gastrointestinal colonization, Multiple locus sequence typing, Endogenic infection, Antibiotic resistance

## Abstract

**Background:**

*Klebsiella pneumoniae* is a leading cause of hospital-associated (HA) infections**.** It has been reported that gastrointestinal colonization (GI) is likely to be a common and significant reservoir for the transmission and infections of *K. pneumoniae* in both adults and neonates. However, the homologous relationship between clinically isolated extraintestinal and enteral *K. pneumoniae* in neonates hasn’t been characterized yet.

**Results:**

Forty-three isolates from 21 neonatal patients were collected in this study. The proportion of carbapenem resistance was 62.8%. There were 12 patients (12/21, 57.4%) whose antibiotic resistance phenotypes, genotypes, and ST types (STs) were concordant. Six sequence types were detected using MLST, with ST37 and ST54 being the dominant types. The results of MLST were consist with the results of PFGE.

**Conclusions:**

These data showed that there might be a close homologous relationship between extraintestinal *K. pneumoniae* (EXKP) and enteral *K. pneumoniae* (EKP) in neonates, indicating that the *K. pneumoniae* from the GI tract is possibly to be a significant reservoir for causing extraintestinal infections.

**Supplementary Information:**

The online version contains supplementary material available at 10.1186/s12866-020-02073-2.

## Background

*Klebsiella pneumoniae* is part of the healthy human microbiome, providing a potential reservoir for infections. It is known that *K. pneumoniae* could asymptomatically colonize the skin, mouth, respiratory, and gastrointestinal tracts (GI). *K. pneumoniae* was detected in approximately 10% of Human Microbiome Project samples collecting from the mouth, nasal cavity, and skin, with an addition of 3.8% stool samples [1]. A 2010 study investigated nasopharyngeal colonization rates for adults and children in Indonesian were 15 and 7%, respectively [2], while another study reported that in the Vietnamese adults, the nasopharyngeal and pharynx colonization rates were 2.7 and 14%, respectively [3]. However, among body sites, GI colonization is likely to be a common and significant reservoir in terms of transmission and infection [4]. In addition, it was reported that *K. pneumoniae* GI colonization rates in hospitalized patients were estimated to be 20 to 38% [5–8]. Furthermore, among intensive care unit (ICU) patients, 48% of screened patients with infection were positive for prior GI colonization [9].

Moreover, *K. pneumoniae* has been recognized as one of the most important opportunistic pathogens in the human gut. Studies have demonstrated that trauma, overuse of antibiotics, and inappropriate diet can destroy the intestinal microecology and decrease probiotics in the gut [10]. These factors could lead to the loss of colonization resistance, allowing for the proliferation of opportunistic pathogens such as *K. pneumoniae* and *Pseudomonas aeruginosa* (PA). These opportunistic bacteria can quickly increase in abundance and has the potential to enter the blood, liver, and lungs, thus leading to enterogenic infections [11, 12]. It was reported that burn injury induces a dramatic dysbiosis of the intestinal microbiome, consequently causing the overgrowth of gram-negative aerobic bacteria, which have the potential to translocate to the extraintestinal sites [13]. Accumulating data [14–16] indicate that *K. pneumoniae* causing late-onset blood infections are of gut origin. However, in fact, we found that gut *K. pneumoniae* might be a reservoir for late-onset respiratory and blood infections.

Therefore, screening the characterization of the carriage *K. pneumoniae* isolates in high-risk patients, could help us predict the probability of potential infections. More importantly, the result of homology analysis of *K. pneumoniae* will provide more evidence. As a result, our study was designed to analyze the relationship between infections and GI colonization among the neonates.

## Results

### Clinical characteristics of the patients

The clinical data of neonates was retrospectively reviewed, and the details were partially shown in Table 1. The 43 strains of different types of specimens were isolated from 21 neonates: feces (*n* = 21, 48.8%), sputa (*n* = 19, 44.2%), and blood (*n* = 3, 7.0%). All patients were treated with two or more antibiotics for a long time (The usage of time of each antibiotic was shown in Table 1), such as mezlocillin/sulbactam (MSU), moxalactam (MOX), ceftazidime (CAZ), piperacillin /tazobactam (TZP), cefotiam (CTF), and meropenem (MEM). All neonates except neonate 1 were discharged after long-stay treatments. Neonate 1 developed multiple organ failure on account of the septicemia caused by *K. pneumoniae*. Considering the probability of the treatment failure, the parents of neonate 1 gave up on further treatments. Complete results were shown in Table 2.

**Table 1:** Clinical characteristics of the *Klebsiella pneumoniae* isolates

Case	Sex	Age	Ward	Diagnosis	Antimicrobial therapy	Use time of antibiotic	Clinical outcome	Isolates	Sample
Patient 1	M	15 days	Neonate	Premature infant HIE	MSU, MOX, MEM		Unchanged	K1	Blood
						15 days		K2	Sputum
								K3	Feces
Patient 2	M	15 days	Neonate	Premature infant HIE	MSU, CAZ, MOX	24 days	Improvement	K4	Sputum
								K5	Feces
Patient 3	M	0 days	Neonate	Respiratory failure	MSU, CAZ, MEM, MOX	50 days	Improvement	K6 K7	Sputum Feces
Patient 4	F	4 days	Neonate	Neonatal pneumonia	MSU, MOX, MEM	56 days	Improvement	K8 K9	Sputum Feces
Patient 5	M	0 days	Neonate	Acute bronchopneumonia	MSU, CAZ, MEM, TZP	75 days	Improvement	K10 K11	Sputum Faces
Patient 6	M	0 days	Neonate	Neonatal encephalopathy	MSU, CAZ, MEM	13 days	Improvement	K12 K13	Sputum Feces
Patient 7	M	0 days	Neonate	Neonatal encephalopathy	MSU, CTF, MEM	13 days	Improvement	K14 K15	Sputum Feces
Patient 8	M	0 days	Neonate	Neonatal pneumonia	MSU, TZP, MEM	16 days	Improvement	K16 K17	Blood Feces
Patient 9	M	0 days	Neonate	Respiratory failure	MSU, TZP, MEM	45 days	Improvement	K18 K19	Sputum Feces
Patient10	M	0 days	Neonate	Neonatal encephalopathy	MSU, TZP, CTF	42 days	Improvement	K20 K21	Sputum Feces
Patient11	M	0 days	Neonate	Neonatal pneumonia	MSU, TZP, CTF, MEM, CAZ	42 days	Improvement	K22 K23	Sputum Feces
Patient12	F	6 days	Neonate	Neonatal pneumonia	MSU, TZP, CTF, MEM	42 days	Improvement	K24 K25	Sputum Feces
Patient13	M	10 days	Neonate	Neonatal pneumonia	MSU, TZP, MEM	29 days	Improvement	K26 K27	Sputum Feces
Patient14	M	5 days	Neonate	Neonatal pneumonia	MSU, CTF, MEM	51 days	Improvement	K28 K29	Sputum Feces
Patient15	F	25 days	Neonate	Neonatal pneumonia	MSU, MOX, CAZ, MEM	41 days	Improvement	K30 K31	Sputum Feces
Patient16	M	0 days	Neonate	Neonatal pneumonia	MSU, TZP, MEM, MOX	51 days	Improvement	K32 K33	Sputum Feces
Patient17	M	30 days	Neonate	Neonatal pneumonia	MSU, CTF, MEM	24 days	Improvement	K34 K35	Sputum Feces
Patient18	M	0 days	Neonate	Respiratory failure	MSU, CAZ, TZP, MEM	31 days	Improvement	K36 K37	Sputum Feces
Patient19	M	17 days	Neonate	Respiratory failure	MSU, CAZ	48 days	Improvement	K38 K39	Sputum Feces
Patient20	M	20 days	Neonate	Respiratory failure	CAZ, TZP, SCF	48 days	Improvement	K40 K41	Sputum Feces
Patient21	M	7 days	Neonate	Respiratory failure	MSU, SCF	11 days	Improvement	K42 K43	Sputum Feces

*M* male; *F* female; *MSU* mezlocillin/sulbactam; *MOX* moxalactam; *MEM* meropenem; *CAZ* ceftazidime; *TZP* piperacillin/tazobactam. *CTF* cefotiam; *SCF* cefperazone/sulbactam

**Table 2:** The ST types, resistant genotypes and resistant phenotypes of the 43 *Klebsiella pneumoniae* isolates K.pneumoniae isolates

Cases	Isolates	STs	Presence of β-lactamase genes	AMP ≥32R^**a**^	PRL ≥128R	AMS ≥32/16R	AMC ≥32/16R	TZP ≥128/4R	KZ ≥8R	CAZ ≥16R	CTX ≥4R	FEP ≥16R	AZT ≥16R	IPM ≥4R	MEM ≥4R	Phenotypes
Patient 1	K1	54	SHV, TEM-1, NDM-1 I ^b^	> 16	> 64	> 16/8	> 16/8	32/4	> 16	> 16	> 32	> 16	< 2	2	2	A^c^
	K2	54	SHV, TEM-1, NDM-1, CTX-M-15 I	> 16	> 64	> 16/8	> 16/8	> 64/4	> 16	> 16	> 32	> 16	> 16	2	> 8	B
	K3	54	SHV, TEM-1, NDM-1, CTX-M-15 I	> 16	> 64	> 16/8	> 16/8	> 64/4	> 16	> 16	> 32	> 16	> 16	2	> 8	B
										> 16^> 16^						
Patient 2^b^	K4	54	SHV, TEM-1, CTX-M-1, CTX-M-15 II	> 16	> 64	> 16/8	> 16/8	> 64/4	> 16	> 16	> 32	> 16	> 16	> 8	> 8	B
	K5	54	SHV, TEM-1, CTX-M-1 II	> 16	> 64	> 16/8	> 16/8	> 64/4	> 16	> 16	> 32	> 16	> 16	> 8	> 8	B
Patient 3	K6	37	SHV, CTX-M-1, CTX-M-15 II	> 16	> 64	> 16/8	> 16/8	> 64/4	> 16	> 16	> 32	> 16	> 16	> 8	< 1	C
	K7	70	SHV, CTX-M-1, CTX-M-15 II	> 16	> 64	> 16/8	> 16/8	> 64/4	> 16	> 16	> 32	> 16	< 2	> 8	> 8	D
Patient 4^b^	K8	37	SHV, CTX-M-1, CTX-M-15 II	> 16	> 64	> 16/8	> 16/8	> 64/4	> 16	> 16	> 32	> 16	> 16	> 8	> 8	B
	K9	37	SHV, CTX-M-1, CTX-M-14, CTX-M-15 II	> 16	> 64	> 16/8	> 16/8	> 64/4	> 16	> 16	> 32	> 16	> 16	> 8	> 8	B
Patient 5	K10	70	SHV, CTX-M-1, CTX-M-15 II	> 16	> 64	> 16/8	> 16/8	> 64/4	> 16	> 16	> 32	> 16	> 16	> 8	> 8	B
	K11	37	SHV, CTX-M-1, CTX-M-15 II	> 16	> 64	> 16/8	> 16/8	> 64/4	> 16	> 16	> 32	> 16	> 16	> 8	> 8	B
Patient 6	K12	37	SHV, CMY-8, CTX-M-1, CTX-M-14, CTX-M-15 III	> 16	> 64	> 16/8	> 16/8	> 64/4	> 16	> 16	> 32	> 16	> 16	> 8	> 8	B
	K13	70	SHV, CTX-M-1, CTX-M-15 II	> 16	> 64	> 16/8	> 16/8	> 64/4	> 16	> 16	> 32	> 16	> 16	> 8	> 8	B
Patient 7	K14	37	SHV, CTX-M-14, CTX-M-15 II	> 16	> 64	> 16/8	> 16/8	> 64/4	> 16	> 16	> 32	> 16	> 16	> 8	> 8	B
	K15	70	SHV, CTX-M-1, CTX-M-15 II	> 16	> 64	> 16/8	> 16/8	> 64/4	> 16	> 16	> 32	> 16	> 16	> 8	> 8	B
				> 16	> 64	> 16/8	> 16/8	> 64/4	> 16	> 16	> 32	> 16		> 8		
Patient 8	K16	37	SHV, CTX-M-1, CTX-M-15, OXA-1 IV	> 16	> 64	> 16/8	> 16/8	> 64/4	> 16	> 16	> 32	> 16	> 16	< 1	< 1	C
	K17	37	SHV, CTX-M-1, CTX-M-14, CTX-M-15 II	> 16	> 64	> 16/8	> 16/8	> 64/4	> 16	> 16	> 32	> 16	> 16	> 8	> 8	B
Patient 9^b^	K18	37	SHV, CTX-M-14, CTX-M-15, OXA-1 III	> 16	> 64	> 16/8	> 16/8	> 64/4	> 16	> 16	> 32	> 16	> 16	> 8	> 8	B
	K19	37	SHV, CTX-M-1, CTX-M-14, CTX-M-15, OXA-1 III	> 16	> 64	> 16/8	> 16/8	> 64/4	> 16	> 16	> 32	> 16	> 16	> 8	> 8	B
Patient 10^b^	K20	1083	SHV, CTX-M-1, CTX-M-15 II	> 16	> 64	> 16/8	> 16/8	> 64/4	> 16	> 16	> 32	> 16	> 16	< 1	< 1	C
	K21	37	SHV, CTX-M-1, CTX-M-15 II	> 16	> 64	> 16/8	> 16/8	> 64/4	> 16	> 16	> 32	> 16	> 16	< 1	< 1	C
Patient 11	K22	37	SHV, CTX-M-1, CTX-M-14, CTX-M-15 II	> 16	> 64	> 16/8	> 16/8	> 64/4	> 16	> 16	> 32	> 16	> 16	> 8	> 8	B
	K23	37	SHV, CTX-M-1, CTX-M-15 II	> 16	> 64	> 16/8	> 16/8	32/4	> 16	> 16	> 32	> 16	> 16	< 1	< 1	C
Patient 12^b^	K24	37	SHV, CTX-M-1, CTX-M-15, TEM-1 II	> 16	> 64	> 16/8	> 16/8	> 64/4	> 16	> 16	> 32	> 16	> 16	> 8	> 8	B
	K25	37	SHV, CTX-M-1, CTX-M-15, TEM-1 II	> 16	> 64	> 16/8	> 16/8	> 64/4	> 16	> 16	> 32	> 16	> 16	> 8	> 8	B
Patient 13^b^	K26	37	SHV, CTXM-M-1, CTX-M-14, CTX-M-15, OXA-1, TEM-1 IV	> 16	> 64	> 16/8	> 16/8	> 64/4	> 16	> 16	> 32	> 16	> 16	> 8	> 8	B
	K27	37	SHV, CTXM-M-1, CTX-M-14, CTX-M-15, OXA-1 IV	> 16	> 64	> 16/8	> 16/8	> 64/4	> 16	> 16	> 32	> 16	> 16	> 8	> 8	B
Patient 14^b^	K28	37	SHV, CTX-M-1, CTX-M-15, TEM-1 II	> 16	> 64	16/8	> 16/8	> 64/4	> 16	> 16	> 32	> 16	> 16	< 1	< 1	C
	K29	37	SHV, CTX-M-1, CTX-M-15 II	> 16	> 64	16/8	> 16/8	> 64/4	> 16	> 16	> 32	> 16	> 16	< 1	< 1	C
Patient 15^b^	K30	37	SHV, CTX-M-1, CTX-M-15, TEM-1 II	> 16	> 64	> 16/8	> 16/8	> 64/4	> 16	> 16	> 32	> 16	> 16	< 1	< 1	C
	K31	37	SHV, CTX-M-1, CTX-M-15 II	> 16	> 64	> 16/8	16/8	> 64/4	> 16	> 16	> 32	> 16	> 16	< 1	< 1	C
Patient 16	K32	1083	SHV, CTX-M-1, CTX-M-15, TEM-1 II	> 16	> 64	> 16/8	> 16/8	> 64/4	> 16	> 16	> 32	> 16	> 16	< 1	< 1	C
	K33	1436	SHV, CTX-M-1, CTX-M-15 II	> 16	> 64	> 16/8	> 16/8	> 64/4	> 16	> 16	> 32	> 16	> 16	< 1	< 1	C
Patient 17^b^	K34	1083	SHV, CTX-M-14, CTX-M-15, TEM-1 II	> 16	> 64	> 16/8	> 16/8	> 64/4	> 16	> 16	> 32	> 16	> 16	< 1	< 1	C
	K35	37	SHV, CTX-M-15 II	> 16	> 64	> 16/8	> 16/8	> 64/4	> 16	> 16	> 32	> 16	> 16	< 1	< 1	C
Patient 18^b^	K36	37	SHV, CTX-M-1, CTX-M-14, CTX-M-15, OXA-1 IV	> 16	> 64	> 16/8	16/8	> 64/4	> 16	> 16	> 32	> 16	> 16	> 8	> 8	B
	K37	37	SHV, CTX-M-14, OXA-1 IV	> 16	> 64	> 16/8	16/8	> 64/4	> 16	> 16	> 32	> 16	> 16	> 8	> 8	B
Patient 19^b^	K38	37	SHV, CTXM-M-1, CTX-M-14, CTX-M-15, OXA-1, TEM-1 IV IVAAA+DA + D	> 16	> 64	> 16/8	> 16/8	> 64/4	> 16	> 16	> 32	> 16	> 16	> 8	> 8	B
	K39	37	SHV, CTX-M-15, OXA-1, TEM-1 IV	> 16	> 64	> 16/8	> 16/8	> 64/4	> 16	> 16	> 32	> 16	> 16	> 8	> 8	B
Patient 20	K40	29	SHV, TEM-1 II	> 16	> 64	> 16/8	> 16/8	> 64/4	> 16	8	2	16	<=4	< 1	< 1	E
	K41	37	SHV, TEM-1 II	> 16	> 64	> 16/8	> 16/8	> 64/4	> 16	> 16	> 32	> 16	> 16	< 1	< 1	C
Patient 21^b^	K42	29	SHV, TEM-1 II	> 16	> 64	> 16/8	> 16/8	> 64/4	> 16	> 16	> 32	> 16	> 16	< 1	< 1	C
	K43	29	SHV, TEM-1 II	> 16	> 64	> 16/8	> 16/8	> 64/4	> 16	> 16	> 32	> 16	> 16	< 1	< 1	C

*ST* sequence type; *AMP* ampicillin; *AMS* ampicillin/sulbactam; *TZP* piperacillin/tazobactam; *AMC* ampicillin/clavulanic acid, *PRL* piperacillin; *KZ* cefazolin; *CAZ* ceftazidime; *CTX* cefotaxime; *FEP* cefepime; *AZT* aztreonam; *IPM* imipenem; *MEM* meropenem

^a^ The breakpoints: ≥32R means resistant breakpoints

^b^This symbol means that antibiotic resistance phenotypes, genotypes and the ST types of the paired isolates from the same patient were consistent

### Antibiotic sensitivity tests

The results of the antibiotic sensitivity tests in this study showed that the isolates were resistant to different classes of antibiotics (Table 2). All the isolates (43/43) were MDR (multiple drug-resistant) (MDR: Resistant to three or more antimicrobial classes [17].). The proportion of carbapenem resistance was 62.8% among all the isolates (Table 2). In addition, we have compared the resistance rates between the EKP and EXKP. Complete results were shown in supplementary file 1.

### Identification of β-lactamase genes and homology analysis of strains

The β-lactamases were divided into four major classes (A to D) by the Ambler scheme [18]. According to the expression of β-lactamase genes, the genotypes were classified into four types (I-IV) I: expressing class A and B β-lactamases; II: expressing class A β-lactamases; III: expressing class A and C β-lactamases; IV: expressing class A and D β-lactamases. The drug resistant phenotypes were divided into five types (A to E) according to the antibiotic sensitive tests. A: resistant to penicillin, penicillin/β-lactamase inhibitors and cephalosporins, sensitive to monobactams and intermediate to carbapenems; B: resistant to penicillin, penicillin/β-lactamase inhibitors, cephalosporins, monobactams and carbapenems; C: resistant to penicillin, penicillin/β-lactamase inhibitors, cephalosporins and monobactams and sensitive to carbapenems; D: resistant to penicillin, penicillin/β-lactamase inhibitors, cephalosporins and carbapenems and sensitive to monobactams; E: resistant to penicillin and penicillin/β-lactamase inhibitors and sensitive to cephalosporins, monobactams and carbapenems. Detailed classifications were shown in Table 2.100% of the isolates (43/43) produced *SHV* (100%), and most produced *CTX-M-15* (79.1%, 34/43) and *CTX-M-1* (69.8%, 30/43). Three isolates were identified as *NDM-1* positive isolates. There were 12 patients (12/21, 57.1%) whose antibiotic resistance phenotypes, genotypes and the ST types were concordant (When the antibiotic resistance phenotypes, genotypes, and the ST types of the strains were concordant, the paired isolates might be homologous.)Complete results were shown in Table 2**.**

**Fig. 1:**
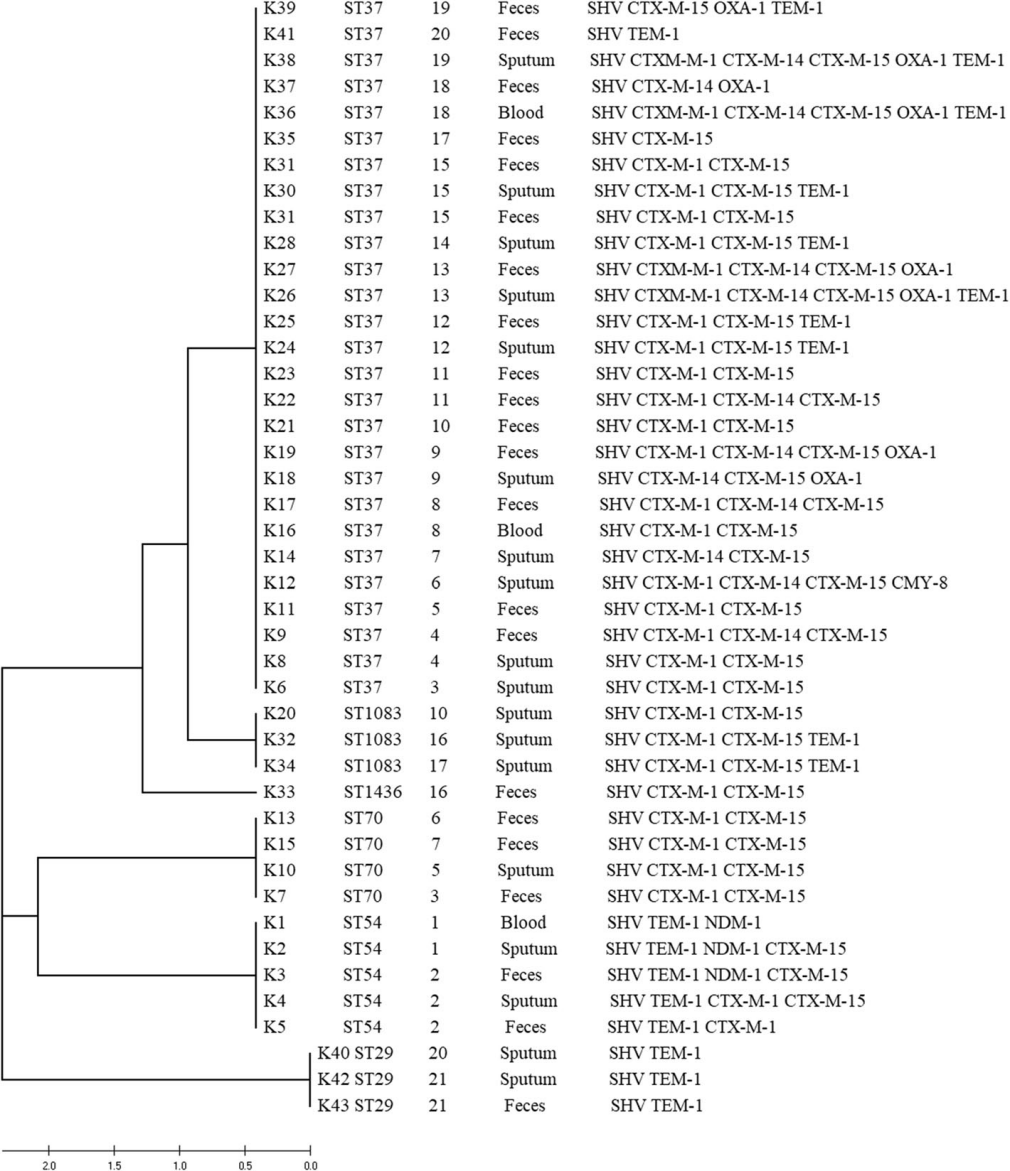
The UMPGA dendrogram, sequence types (STs), and genotypes of 43 *Klebsiella pneumoniae* isolates from 21 patients. The tree shows that ST37 and ST1083 were related. In our previous study, ST37 and ST1083 belong to the same clone complex CC37 [19]

**Fig. 2:**
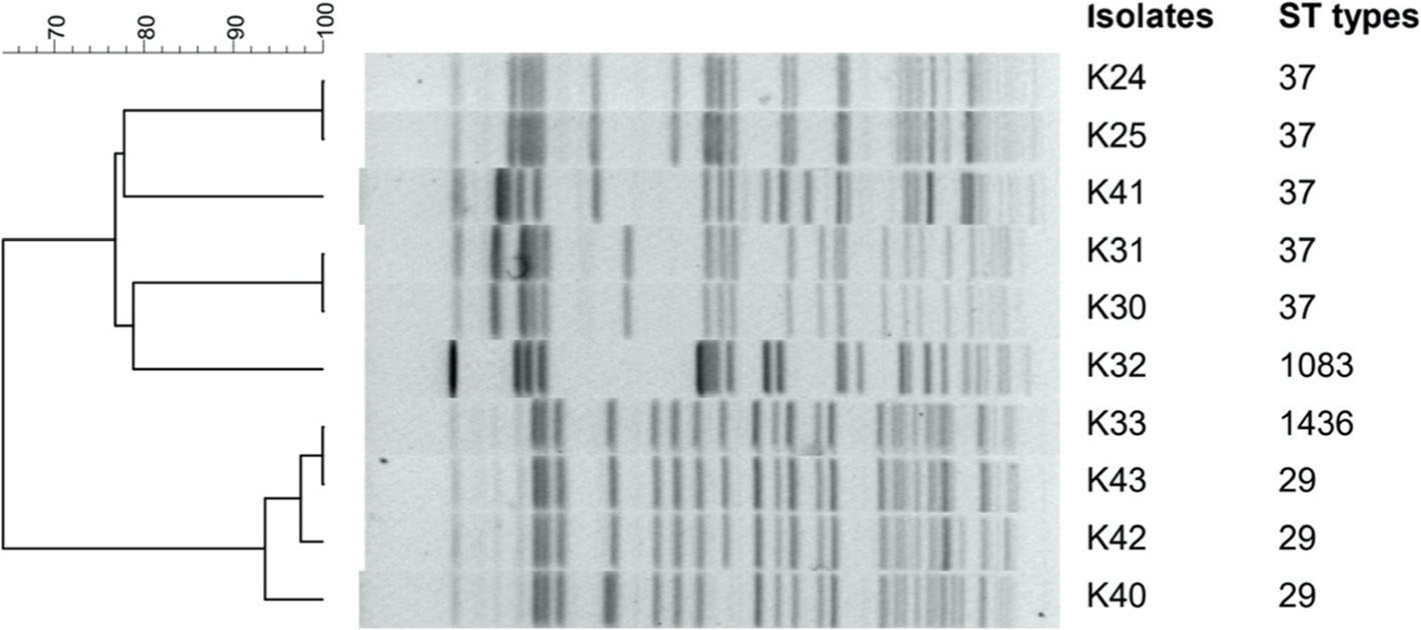
Dendrogram comparing PFGE profile of *K. pneumoniae* (patients 12, 15 and 21) together with the results of MLST. The PFGE shows that the paired isolates from patients 12, 15 and 21 had identical and > 90% similarity in PFGE patterns

The STs of the isolates were determined and numbered using the international database of the Institute Pasteur website, which showed an immense diversity with the results presented in Table 2. The isolates were distributed in six types of STs (ST37, 54,70, 29,1083,1436), among which ST37 and ST54 were the most frequently seen STs. Besides, our data indicated that the ST37 was the main ST type in both the extraintestinal and enteral isolates. The concatenated sequences of all seven loci were used to draw a phylogenetic tree. The results showed that ST37 and ST1083 were homologous, which belong to CC37 clone complex [19]. The result of PFGE also demonstrated that ST37 and ST1083 were homologous. Complete results were shown in Fig. 1 and Fig. 2.

### Pulsed-field gel electrophoresis analysis

Among five patients, the pulsed-field gel electrophoresis analysis (PFGE) showed the paired isolates from patients 12,15 and 21 had identical and > 90% similarity in PFGE patterns (Fig. 2).

## Discussion

*K. pneumoniae* is known as the common cause of respiratory tract infections, urinary tract infections (UTIs), and bloodstream infections (BSIs) [20]. *K. pneumoniae* typically colonize human mucosal surfaces, including nasopharynx and GI tract. The colonization rate varies among different body sites, and also is different between the community-acquired (CA) *K. pneumoniae* and the hospital-acquired (HA) *K. pneumoniae*. It is estimated that the rate of CA nasopharynx colonization was about 11%. The rate in adults is typically higher than that in children [20]. However, the rate of HA nasopharynx colonization is slightly higher, up to 19% [21]. Compared to the nasopharynx, the CA GI colonization rate is estimated to be around 3.9 ~ 5.9% [9]. Furthermore, the HA GI colonization rate varies from 23 to 30% [22, 23]. It was reported that the GI carriage of *K. pneumoniae* was related to the subsequent HA infections [6]. In 2017, a study which explored the association between GI colonization and infections. Showed that the rate of *K. pneumoniae* infections was much higher for the GI colonization patients compared with the patients who were culture-negative (16% vs 3%) [9]. However, for the neonates, intestinal colonization occurred immediately after birth [24]. When some pathogens colonize the gut, it might result in the later subsequent infections. Compared to the neonates who were non-colonized, the likelihood of the colonized-neonates developing subsequent infections was remarkably higher (24.8% VS 1.9%). The percentages of the nasopharynx and GI *K. pneumoniae* colonization were respectively 29 and 36.8% in the hospitalized neonates [25]. Furthermore, a study showed that the GI *K. pneumoniae* could invade and penetrate the intestinal epithelium, which indicated that GI *K. pneumoniae* could cause extraintestinal infections. This transcellular translocation mechanism is exploited by *K. pneumoniae* strains from the gut caused systematic infections by this transcellular mechanism [26]. Although there was a close relationship between colonization and infections, the homologous relationship between the GI colonized isolates and extraintestinal isolates has not been reported yet.

In our study, all the isolates (43/43) were MDR *K. pneumoniae*, and 27 strains were resistant to carbapenems with a drug resistance rate of 62.8%. The proportion was moderately higher than 54% in adult that published by World Health Organization [27], while considerably higher than the proportions of 24.7 and 29.8% found in previous studies in the neonates [28, 29].

One hypothesis indicating that GI colonization was likely to be a significant reservoir in terms of transmission and infections [4]. Furthermore, some drug-resistant genes which were mediated by plasmids could be acquired or lost during bacterial translocation [20]. Based on this hypothesis, the drug-resistant phenotypes might be affected by the loss or acquisition of the β-lactamase genes. In our study, the CTX-M-1, CTX-M-14, CTX-M-15, and TEM-1 were expressed differently between feces and other samples. These genes belong to plasmid-mediated ESBLs [18]. In this case, the drug resistant genotypes and phenotypes were divided into different groups as per the antibiotic sensitivity tests and the expressions of the β-lactamase genes. The results showed that there were 12 patients (12/21, 57.1%) whose paired isolates might be homologous. The data demonstrated that the GI tract might be a significant reservoir for causing extraintestinal infections.

The majority of the isolates were resistant to β-lactam antibiotics. The resistance observed in the present study might be attributed to the expression of resistance genes such as β-lactamase genes. *NDM-1* appeared in 7% of isolates, which was first identified in 2006. After it was first identified, it was predominantly found in *K. pneumoniae* and *E. coli.* Since 2010, the bacteria producing NDM-1 had been reported worldwide. In China, NDM-1 producing *K. pneumoniae* has been frequently reported in neonates [30, 31]. The STs of *blaNDM-1-*producing *K. pneumoniae* mainly included ST11, ST16, ST20, ST37, ST70, ST147, and ST1419 [32–35]. But our data indicated that the ST54 was the only NDM-1 producing type. In most cases, bacteria with NDM-1 were resistant to almost all antibiotics. Moreover, the dissemination has been facilitated by horizontal gene transfer. That being so, reliable detection and surveillance are of great importance in preventing the clonal outbreaks. Although these isolates showed high drug resistance and high rates of resistance genes, just one neonate (patient 1) acquired a poor prognosis upon treatment with antibiotics.

To confirm whether the isolate pairs were homologous or not, a UMPGA tree was drawn by employing MEGAX to further analyze the homology among the different isolates from the same patients. Excluding the completely concordant strains, the analysis of the homology among ST37 and ST1083 should be confirmed. According to the analyses, ST37 and ST1083 were in the same cluster (two alleles of the 7 housekeeping genes differed), concluding that the two were closely related and the results validated a great deal of our previous research [19]. The PFGE also indicated that ST37 and ST1083 were homologous. Moreover, our data indicated that ST37 were the main epidemic clones in the Neonate Ward, which showed consistency with what found in other studies [19, 30]. It is discovered that ST37 are presumably to be a potential high-risk MDR *K. pneumoniae* clonal lineage [36]. In our study, the results of MLST were consist with PFGE. Furthermore, it is reported that carriage of carbapenem-resistant *K. pneumoniae* (CRKP) in the GI tract may precede and possibly serve as a source for subsequent clinical infections in approximately 9% of carriers [37, 38]. And these carriers may act as a significant reservoir for the dissemination of CRKP in the healthcare facilities [39–41]. Combined with our study, active surveillance for detecting CRKP colonization is critical for preventing the CRKP from spreading. Besides, according to the guidance of CDC for control of Carbapenem-Resistant Enterobacteriaceae (CRE), screening rectal cultures of CRE is an important strategy for CRE prevention [42].

The main strength of our study is the use of multiple approaches to characterize the isolates and their similarity to one another in the neonates. However, there are several limitations. First, neonatal cases are difficult to collect, only 21 neonatal patients were collected for analyzing. Second, because of the limitation of experimental conditions, only 10 paired strains from 5 patients were selected randomly for PFGE.

## Conclusion

In this study, we found there was an apparently close phylogenetic relationship between the extraintestinal and enteral strains. This conclusion is a reminder that *K. pneumoniae* which colonizes in the intestine can also induce infections in other parts of the body. Once the Amp C, KPC, and *NDM-1* genes are successfully transferred, acquired resistance will potentially cause severe infections. Therefore, the hospital should screen the CRKP which colonized in the gut to limit and prevent current and future outbreaks.

## Methods

### Bacterial strains and clinical characteristic

Samples isolated from the feces, sputa, and blood were collected from the neonates infected with *K. pneumoniae.* All the sputum samples were collected from the neonates who were diagnosed with neonatal pneumonia, acute bronchopneumonia, and bronchitis. Diagnoses were made based on both clinical and radiologic findings. The strains isolated from the same patient were paired. Forty-three isolates of *K. pneumoniae* were collected from feces, sputa, and blood of 21 neonates. All the neonates were admitted to the Second Xiangya Hospital of Central South University, China, from July 2014 to April 2015. All the data of the neonates were collected by chart review from the hospital’s unified electronic database. These isolates were identified by using the BD Phoenix 100 Automated Microbiology System (BD Diagnostic Systems, MD, USA). *Escherichia coli* ATCC 25922 and *K. pneumoniae* ATCC 700603 were used as quality control strains.

### Antibiotic susceptibility testing

All bacterial isolates were subjected to antibiotic sensitivity tests using the agar dilution method following the standard antibiotic susceptibility test chart from the CLSI guidelines [43]. The results were interpreted by measuring the minimum inhibitory concentrations (MICs) which were determined as the lowest concentration of antibiotics at which the strains showed no visible growth after overnight incubation at 37 °C. The isolates resistant to carbapenems were verified with the Kirby-Bauer/disk diffusion method following the CLSI guidelines [43].

### PCR and sequencing for resistant genes

Genomic DNA from the isolates was prepared for PCR and genetic analyses using the TIAN amp Bacterial DNA Kit (Tian Gen Biotech, Beijing, Co., Ltd.). The β-lactamase antibiotic resistance genes which were prevalent in *K. pneumoniae* were mainly detected (including NDM-1, KPC-2, OXA-1, OXA-2, OXA-9, OXA-48, OXA-181, CTX-M-1, CTX-M-2, CTX-M-8, CTX-M-14, CTX-M-15, CMY-4, CMY-8, TEM-1, and SHV; Table 3). These resistance genes were screened through PCR assays, and the PCR products were sent to Sangon Biotech (Shanghai)Co., Ltd. for sequencing analysis. The entire sequence of each gene was compared to the sequences in the Gen-Bank nucleotide database at http://www.ncbi.nlm.nih.gov/blast/.

**Table 3:** Primers used in this study

Primers	Primers sequence (5′-3′)	Annealing temperature (°C)	Length of products (bp)	Ref.
NDM-1	Sense: 5′-CCGCAACCATCCCCTCTT-3′ Anti: 5′-CAGCACACTTCCTATCTC-3′	53	888	This study
KPC-2	Sense: 5′-GGCACTTTTCGTTCCA-3′ Anti: 5′-ATGATTTTCAGAGCCTTACT-3′	52	1003	This study
OXA-1	Sense: 5′-CTGTTGTTTGGGTTTCGCAAG-3′ Anti: 5′-CTTGGCTTTTATGCTTGATG-3′	55	440	This study
OXA-2	Sense: 5′-TAAGCAACACCGACAGG-3′ Anti: 5′-TCGTGATGAGTTCCAGAT-3′	51.2	879	This study
OXA-9	Sense: 5′-ACAGCGGAGCAATGAAG-3′ Anti: 5′-CGACAAAGCGTAGAAGAAAC-3′	52.6	549	This study
OXA-48	Sense: 5′-TTTTCCTGTTTGAGCACT-3′ Anti: 5′-TACCCGCATCTACCTTT-3’	50	586	This study
OXA-181	Sense: 5’-5CGTTATGCGTGTATTAGC-3′ Anti: 5′-CACTTCTTTTGTGATGGC-3’	51	775	This study
CTX-M-1	Sense: 5’-CAGCGCTTTTGCCGTCTAAG-3′ Anti: 5′-GGCCCATGGTTAAAAAATCACTGC-3’	60	945	[44]
CTX-M-2	Sense: 5’-CTCAGAGCATTCGCCGCTCA-3′ Anti: 5′-CCGCCGCAGCCAGAATATCC-3’	61.5	843	[44]
CTX-M-8	Sense: 5’-ACTTCAGCCACACGGATTCA-3′ Anti: 5′-CGAGTACGTCACGACGACTT-3’	52.5	1024	[44]
CTX-M-14	Sense: 5’-GCAGATAATACGCAGGTG-3′ Anti: 5′-GCTGGGTAAAATAGGTCAC −3’	55.1	640	This study
CTX-M-15	Sense: 5’-ATTAGAGCGGCAGTCGG-3′ Anti: 5′-AAGGAGAACCAGGAACCAC-3’	55.1	883	This study
CMY-4	Sense: 5’-GCCGTTGCCGTTATCTAC-3′ Anti: 5′-CCAATGCCACTTTGCTGT-3’	55.2	796	[45]
CMY-8	Sense: 5’-AGCGGTAAACGAGTGAG-3′ Anti: 5′-AGTAATGCCCTTTGTGG-3′	52	1042	[45]
TEM-1	Sense: 5′-TTCGTGTCGCCCTTATTC-3′ Anti: 5′-ACGCTCGTCGTTTGGTAT-3′	55	512	This study
SHV	Sense: 5′-GCCTTTATCGGCCTTCACTCAAG-3′ Anti: 5′-TTAGCGTTGCCAGTGCTCGATCA-3′	60	898	[44]

### Multiple locus sequence typing

The MLST assay was performed as previously described [43]. Briefly, seven *K. pneumoniae* housekeeping genes (*infB, tonB, pgi, gapA, phoE, rpoB,* and *mdh*) were amplified and sequenced. Alleles and STs were assigned using the *K. pneumoniae* MLST database (http://bigsdb.web.pasteur.fr/klebsiella/klebsiella.html).

### Phylogenetic relationship

The products of the housekeeping genes were compared and analyzed by utilizing the program BLAST. To explore the phylogenetic relationship among the isolates, the seven loci (*rpoB, gapA, mdh, pgi, infB, phoE*, and *tonB*) of each isolate were concatenated and aligned using the Clustal X program. An evolutionary tree for the data set was formed by the UMPGA tree using the software MEGA X. The stability of the phylogenetic relationship was evaluated by bootstrap analysis based on 1000 replicates [46]. The tree was drawn to scale, with branch lengths in the same units as those of the evolutionary distances used to infer the phylogenetic tree [47].

### PFGE

We performed PFGE analysis using Bio-Rad syste m[48]. First, bacterial suspension was prepared, and then the restriction enzyme XbaI was used. Second, the electrophoretic gel was imprinted, and stained with ethidium bromide. Finally, electrophoretic images were analyzed with the software BioNumerics (Applied Maths, Inc.). A similarity coefficient > 80% was selected to define a major cluster.

### Statistical analysis

All data were analyzed with SPSS 19.0 statistical software. Categorical variables were evaluated by the Fisher’s exact test. Values were presented as percentages of the group from which they were derived (categorical variables). A *p* value of < 0.05 was considered statistically significant. Bio Numerics 5.10 software was used for PFGE.

## Supplementary Information

**Additional file 1.**

## Data Availability

The datasets used and/or analyzed during the current study are available from the corresponding author on reasonable request.

## Abbreviations

HA: Hospital-associated
GI: Gastrointestinal colonization
PCR: Polymerase chain reactions
MLST: Multiple Locus Sequence Typing
EXKP: Extraintestinal *K. pneumoniae*
EKP: Enteral *K. pneumoniae*
STs: ST types
ICU: Intensive care unit
PA: *Pseudomonas aeruginosa*
MICs: Minimum inhibitory concentrations
MSU: Mezlocillin/sulbactam
MOX: Moxalactam
CAZ: Ceftazidime
TZP: Piperacillin /tazobactam
CTF: Cefotiam
MEM: Meropenem
MDR: Multiple drug-resistant
PFGE: Pulsed-field gel electrophoresis analysis
UTIs: Urinary tract infections
BSIs: Bloodstream infections
CA: Community-acquired
HA: Hospital-acquired
CRKP: Carbapenem-resistant *K. pneumoniae*
CRE: Carbapenem -Resistant Enterobacteriaceae
NDM: New Delhi metallo-beta-lactamase
KPC: *Klebsiella pneumoniae* carbapenemase
OXA: Oxacillinase
CTX-M: Cefotaximase
CMY: Cephamycinase
TEM: Temoneira
SHV: Sulfhydryl Variable
