# Correction to: Homology analysis between clinically isolated extraintestinal and enteral *Klebsiella pneumoniae* among neonates

**DOI:** 10.1186/s12866-021-02100-w

**Published:** 2021-02-02

**Authors:** Chun-mei Chen, Min Wang, Xian-ping Li, Peng-ling Li, Jing-jing Tian, Kan Zhang, Can Luo

**Affiliations:** 1 Department of Laboratory Medicine, The Second Xiangya Hospital, Central South University, 139 Renmin Road, Changsha, 410011 Hunan China; 2 Department of Laboratory Medicine, The Fifth Xiangya Hospital, Central South University, Changsha, Hunan China; 3 Department of Laboratory, Medicine, Jiangxi Cancer Hospital, Nanchang, Jiangxi China

**Correction to: BMC Microbiol 21, 25 (2021)**

**https://doi.org/10.1186/s12866-020-02073-2**

Following publication of the original article [1], we were notified that the Funding note should be updated.

- Originally published Funding:

“This work was supported by the Hunan Science and Technology Innovation Project, China (grant number 2017SK50122) and the Central South University New Medical Technology Project.”

- Corrected Funding:

“This work was supported by the Hunan Science and Technology Innovation Project, China (grant number 2017SK50122) and the New medical technology project of the Second Xiangya Hospital.”

Secondly, affiliation 1 (Department of Laboratory Medicine, The Fifth Xiangya Hospital, Central South University, Changsha, Hunan, China) should be removed for the author Chunmei Chen.

The original article has been corrected.
