# Supplementary material for: Homology analysis between clinically isolated extraintestinal and enteral Klebsiella pneumoniae among neonates
Source: BMC Microbiol. 2021 Jan 11;21:25. doi: 10.1186/s12866-020-02073-2 (PMC7802202; doi:10.1186/s12866-020-02073-2)
Supplement: Supplementary file 1 — Additional file 1. [file 12866_2020_2073_MOESM1_ESM.docx]

The comparations of resistance rates between EKP and ERKP isolates

| Antibiotics | EKP(resistance rate%) | EXKP(resistance rate%) | P value |
| --- | --- | --- | --- |
| AMP | 22(100) | 21(100) | *P>0.05* |
| PRL | 22(100) | 21(100) | *P>0.05* |
| AMS | 21(95.5) | 20(95.2) | *P>0.05* |
| AMC | 20(90.9) | 20(95.2) | *P>0.05* |
| TZP | 21(95.5) | 20(95.2) | *P>0.05* |
| KZ | 22(100) | 21(100) | *P>0.05* |
| CAZ | 22(100) | 20(95.2) | *P>0.05* |
| CTX | 22(100) | 20(95.2) | *P>0.05* |
| FEP | 22(100) | 20(95.2) | *P>0.05* |
| AZT | 22(100) | 18(85.7) | *P>0.05* |
| IPM | 12(54.5) | 11(47.8) | *P>0.05* |
| MEM | 13(59.1) | 12(57.1) | *P>0.05* |
